# “DiscoverU”: Formative Development and Mixed‐Methods Evaluation of an Afterschool Health and Well‐Being Mentoring Program for Adolescents

**DOI:** 10.1002/jcop.70137

**Published:** 2026-08-05

**Authors:** Katherine R. Arlinghaus, Kristen Stuenkel, Jodi Gadient, Adrianna N. Bell, Lenora P. Goodman, Nancy E. Sherwood, Barbara J. McMorris

**Affiliations:** ^1^ University of Minnesota School of Public Health Division of Epidemiology & Community Health Minneapolis Minnesota USA; ^2^ Columbia Heights Public School District Columbia Heights Minnesota USA; ^3^ University of Minnesota School of Nursing Minneapolis Minnesota USA

**Keywords:** adolescent, afterschool, mentoring, mindful eating, physical activity, school health, social‐emotional learning

## Abstract

This manuscript describes the (1) development of the “DiscoverU” afterschool mentoring program and (2) formative evaluation of its feasibility, acceptability, and initial impact on adolescents' physical activity (PA), interpersonal skills, and social connectedness. We used a convergent mixed‐methods design with quantitative data from adolescents (*n* = 32) and qualitative data from adolescents (*n* = 14), mentors (*n* = 14), facilitators (*n* = 3), and school staff (*n* = 3). We assessed pre–post changes physical activity, mindful eating, and social‐emotional skills with paired Wilcoxon tests and used content analysis to identify themes. DiscoverU was highly acceptable and demonstrated initial impacts on PA days/week, peer cooperation, and social connection. Qualitative data indicated adolescents' experience with mentors was important to these outcomes. However, feasibility was limited by inconsistent attendance with 44%–52% of adolescents receiving the intended dose. DiscoverU's novel use of mentoring to integrate physical and mental health promotion was well‐liked by all involved parties. Strategies to enhance consistent adolescent attendance are needed to increase program feasibility.

## 1 Introduction

Adolescents face many challenges coming out of the COVID‐19 pandemic. Already rising rates of adolescent suicide, depression, and anxiety further worsened during and after the pandemic (Ivey‐Stephenson et al. 2020; Jones et al. 2022; Warhadpande et al. 2023; Wilson and Dumornay 2022). These declines in mental health occurred alongside increases in cardiovascular risk factors such as obesity, insulin resistance, and physical inactivity (Lange et al. 2021; Warhadpande et al. 2023), leaving schools with the daunting task of addressing pandemic‐related declines in academic achievement amongst adolescents struggling with both their mental and physical health. Schools in communities with fewer resources are disproportionately challenged with addressing these declines as families in these communities had less access to support during and after the pandemic, which may have widened already existing academic and health disparities (Anders et al. 2023; Oberg et al. 2022; White et al. 2021).

Afterschool programs are well‐positioned to help address these challenges; they can extend social and physical health benefits of the school day into a time in which adolescents are likely to otherwise be alone and involved in sedentary, screen‐time behaviors (Haycraft et al. 2020). School‐based afterschool programs provide an environment to reach adolescents from disadvantaged or marginalized backgrounds at heightened risk for adverse health outcomes (White et al. 2021). However, infrastructural support for these programs and staff turnover are ongoing challenges to providing quality afterschool programming, particularly in low‐resource communities (Frazier et al. 2019). These challenges were exacerbated by the pandemic. In Fall of 2021, 51% of out‐of‐school‐time providers reported being extremely concerned about finding staff to hire (Afterschool Alliance 2022). Teachers who usually stayed after school to lead afterschool programming experienced burnout and lacked capacity to assist with programming outside of school hours. This staff shortage contributed to increased afterschool programming costs (Afterschool Alliance 2022), which can worsen disparities in the availability of quality programming to adolescents in lower‐resourced communities and those from racial and ethnic minority groups. Schools in these communities need support to help provide quality, affordable programming to adolescents.

### 1.1 Integrating Social‐Emotional and Physical Health in Afterschool Programming for Adolescents

Afterschool programs are uniquely suited to deliver integrated social‐emotional and physical health opportunities through empowerment‐based approaches (Zimmerman 1995; Zimmerman and Eisman 2017), but to our knowledge, no afterschool programs exist that explicitly integrate nutrition education, physical activity, and social‐emotional learning for adolescents. Addressing these areas of health simultaneously may appeal to time and resource‐constrained schools as an efficient way to improve adolescents' physical and emotional well‐being. Yet, most school‐based interventions focus on only one area of health at time, disregarding how health behaviors are interconnected and tend to cluster (Bonnesen et al. 2020). Addressing either social‐emotional or physical health can create synergies for the other area of health (Kalata et al. 2026; Wade et al. 2026). For example, social connections help explain some of the mental health benefits of physical activity (Lubans et al. 2016). Social connection and support are also critical to youth engagement in physical activity (e.g., peer acceptance, peer affiliation, peer modeling of physical activity, and peer praise for physical activity) (Fitzgerald et al. 2012). In turn, physical activity can facilitate social connections by providing opportunities to learn and practice social skills that deepen the quality of social relationships (Eime et al. 2013). As another example, self‐awareness of recognizing internal cues for hunger and fullness and emotional awareness of reasons for eating like stress or boredom are components of mindful eating approaches to improve dietary consumption (Fernández‐Demeneghi et al. 2026).

Adolescence is a critical developmental period for interventions that integrate physical health with social‐emotional learning. Developmentally, adolescents seek greater autonomy from parents. The greater level of responsibility youth take for their health behaviors during adolescence increases the likelihood that positive changes in physical activity and eating behaviors will be sustained in adulthood (Bélanger et al. 2015). However, adolescents also highly value peer perceptions and peer influence is especially strong when peers are popular or perceived as more mature (Allen et al. 2014; Tucker et al. 2014). Healthy behaviors are often seen as undesirable among peer groups in adolescence. For instance, adolescents report eating healthfully as “socially risky” and worry about being ridiculed for liking specific kinds of foods (Ragelienė and Grønhøj 2020). Similarly, adolescent girls report physical activity to be “uncool” and deprioritized over more mature activities like “going out” (Slater and Tiggemann 2010). Social‐emotional skills like positive self‐awareness and interpersonal skills are critical to support healthy decision making in the face of such social pressures (Botvin and Griffin 2004). Intervention during adolescence requires social‐emotional skill building to support healthy decision making to overcome potential negative peer influences and the promotion of positive peer influences to increase the appeal of healthy behaviors.

### 1.2 Cross‐Age Group Mentoring to Make Health Interventions Appealing

Making health behavior change appealing to adolescents is challenging. Adolescents perceive their concerns to be better understood by peers, are more likely to model behaviors after peers than adults, and feel a sense of responsibility and loyalty to social networks (Laursen and Veenstra 2021). Cross‐age mentorship interventions (e.g., older teen or college‐age students mentoring adolescents) have been shown to improve youth physical, psychological, and social health (Burton et al. 2022). Having recently gone through similar experiences, young adult mentors are relatable, provide advice, and offer adolescents social prestige by association (Bolshakova et al. 2018; Smith 2011). College‐age mentors can help overcome the credibility issue many peer‐led programs face of propagating an “adult health agenda” by being young enough to develop a peer‐like connection, but old enough for adolescents to perceive the health‐promoting agenda as genuine and appealing (Christensen et al. 2020). Group mentoring, in which mentors work with multiple adolescents in a specific site‐based setting, has been shown to have similar if not better outcomes for youth mentees (DuBois et al. 2011). Mentors can help adolescents navigate peer interactions and model healthy behaviors in the larger group setting.

Group‐based mentoring in the context of a health intervention also provides natural opportunities for mentors and adolescents to engage in goal‐directed activities, which is a recommended practice to move beyond expecting positive youth outcomes to occur directly as the result of a caring relationship (Luo and Stoeger 2023). Despite these benefits, most group school‐based mentoring programs are focused on higher risk youth (e.g., Joseph and Kuperminc 2022; Kuperminc et al. 2020; Weiler et al. 2019). This type of model, however, is especially promising for the adolescent afterschool context as a universal intervention because youth self‐select into small groups or classes based on their interests—a key strategy that empowers youth in the school setting (Zimmerman 1995). Finally, a health promotion program incorporating group mentoring may benefit not only individual youth but also the school itself, by establishing social networks into the school district that enhance connections to one's school and community (Portwood et al. 2005).

### 1.3 University‐K12 Partnership & Intervention Development

Building upon previous literature, we established a University‐K12 school partnership to develop an afterschool program that used cross‐age mentoring to try to make social‐emotional and physical health appealing to adolescents. Beginning in Fall of 2021, we worked with an urban school district to develop and pilot a health‐based afterschool program. Importantly, the school district's 21st Century Community Learning Center (CCLC) funding provided critical infrastructure for a variety of afterschool offerings, within which a new curriculum could be developed and piloted. Our research team conducted an initial needs assessment in which students and school staff endorsed a gap in available programming that addressed both physical and mental health concerns of adolescents in middle and high school without over‐extending school personnel. We then developed an afterschool mentoring program that emphasized social connections to improve mental and physical health, relying on strengths of our partnership to impact multiple areas of youth development and health simultaneously (DuBois et al. 2011). Specifically, the program trained college‐age mentors to lead and participate in structured activities with middle and high school students. The activities were designed to explicitly integrate social‐emotional learning, physical activity, and nutrition. We described this program to school staff and students at a school wellness committee meeting, at which time a high school student named it “DiscoverU” to reflect the premise of the curriculum: to better understand yourself and learn ways to live healthfully. The “U” in the name was also a nod to the partnership between the university and local school district. This type of partnership is consistent with the Whole School, Whole Community, Whole Child model (Centers for Disease Control and Prevention 2020), which emphasizes how leveraging expertise of community organizations, such as a local university, can boost the capacity of schools and afterschool programs to holistically support child health. Specifically in the afterschool space, schools are encouraged to partner with other organizations in the community to increase the quality and sustainability of afterschool options that can be offered to students. In fact, partnership is one of the key indicators of a quality afterschool program (de Kanter et al. 2003). Partnerships with universities and K12 schools are common as both institutions share values of education. Our partnership was rooted in being mutually beneficial for both organizations. Specifically, college students desire experiential learning opportunities and K12 schools are often looking for volunteers to assist with programming and be additional caring adults for youth. Our partnership was additionally rooted in shared values of improving the health of young people. These shared values and mutually beneficial arrangements were the cornerstone of our partnership. On the continuum of community‐engaged research, our partnership most closely resembled a community advisor model with school staff being involved at all aspects of the research process (Marrone et al. 2022).

### 1.4 The Present Study

This study aimed to (1) describe the formative development of the DiscoverU mentoring program; (2) evaluate the program's feasibility (e.g., recruitment and retention of adolescent participants) and acceptability (e.g., belonging); and (3) begin to explore the impact on adolescents' physical activity, interpersonal skills (e.g., cooperative behaviors), and social connection with mentors. We used a convergent mixed‐methods design to evaluate DiscoverU. This methodology integrates both qualitative and quantitative data that are collected in parallel to provide a more comprehensive evaluation of the intervention rather than qualitative or quantitative data can provide alone (Creswell and Clark 2017). A separate manuscript will focus on the college‐student mentors.

## 2 Methods

### 2.1 Participants and Setting

The research team partnered with the community education department of a nearby school district that serves a racially and ethnically diverse student body of whom over 75% are eligible for free or reduced‐price school meals (i.e., family household income ≤185% poverty threshold). Additional demographic characteristics of study participants are provided in the results section. Through 21st CCLC funding in the school district, the district offers adolescents free enrollment in a variety of 60‐min afterschool classes (e.g., weightlifting, chess, knitting, video games, art, etc.). DiscoverU was offered as one of the afterschool class options. To increase the external validity of the pilot program, DiscoverU recruitment and enrollment procedures for middle and high school students mirrored those used by all other afterschool classes: flyers, video announcements, word of mouth, and a variety of in‐person recruitment events (i.e., recruitment tables at open houses, in the common areas before school in the morning and during lunchtime, and DiscoverU activity demonstrations during health classes).

Any middle or high school student in the district was eligible to participate in DiscoverU. Once signed up for DiscoverU, students and their parents/guardians received information about enrolling in the pilot study. Written parental consent and adolescent assent were required to participate in surveys and focus group data collection. Students were welcome to participate in DiscoverU without participating in the study (i.e., data collection). A total of 63 students participated in the program, but only 32 participated in the study. Mentors also provided written consent to participate in surveys and focus groups. Adolescents received a $10 gift card if they completed two surveys (pre‐ and post‐surveys) and another $10 gift card if they participated in a focus group. The University of Minnesota Institutional Review Board approved these research procedures in accordance with CDC's policies and procedures.

### 2.2 Program Development

#### 2.2.1 Overview

The DiscoverU program was developed in three stages: (1) needs assessment and partnership development; (2) program development; and (3) implementation and revisions. We implemented the initial version of the program in Spring of 2022 (March–May) and revised the program over the summer. We implemented the revised program in Fall of 2022 (October–December) and again in Winter of 2023 (January–March) and report on the evaluation of this revised program in this manuscript.

#### 2.2.2 Stage 1: Needs Assessment & Partnership Development

We used an adapted version of the Healthy Eating and Physical Activity (National Afterschool Association's HEPA) self‐assessment tool (National Afterschool Association 2018) for out‐of‐school time programs to conduct an initial needs assessment in Fall 2021. This needs assessment was conducted via walkthroughs of the school and conversations with the school wellness committee which included administration, teachers, and students. Additionally, university team members met with afterschool staff 1–2 times each month to troubleshoot and continue to learn school needs throughout each phase of implementation. University staff volunteered at school events (e.g., vaccine clinics and open houses), attended school functions (e.g., sporting events and plays), helped lead health‐related activities at school staff professional development days, and completed youth mental health trainings alongside school staff. These activities helped university staff better understand school culture, build trust, contribute as partners, and clarify school priorities and needs (Israel et al. 1998).

#### 2.2.3 Stage 2: Development—Theoretical Basis

The DiscoverU curriculum was grounded in social cognitive theory (Bandura 2004), which proposes that behavioral factors (i.e., skills needed to perform a behavior), personal cognitive factors (i.e., self‐efficacy and outcome expectations), and environmental factors reciprocally and interactively influence health behaviors. During structured time after school, DiscoverU influenced students' environment by providing students with a nutritious snack, structured physical activity opportunities, and a supportive, safe emotional climate. To promote this type of climate, mentors were trained to be consistent for youth by being at program when scheduled, greeting all adolescents and accepting them for themselves when they came to program, and responding supportively, without judgment, when adolescents did not know something, did poorly in a game/skill, or expressed a different opinion from others. To further influence the social environment and youth self‐efficacy for physical activity, college‐age mentors offered social connection, modeling, vicarious experience, and encouragement to engage students in activities, including goal‐setting. The explicit integration of social‐emotional learning with physical activity and nutrition activities was meant to promote skill building (e.g., behavioral factors) and self‐awareness (e.g., personal cognitive factors) to help adolescents improve their health behaviors and overall mental and physical health. We focused on two main areas of social‐emotional skill development: self‐awareness (identifying strengths and values; emotional regulation) and interpersonal skills (communication, cooperative behaviors, and problem‐solving).

#### 2.2.4 Stage 2: Development—Curriculum

Through our needs assessment and partnership building activities, needs related to physical activity, nutrition, and social‐emotional learning were all identified. For example, teachers reported noticeable declines in student physical fitness and interpersonal skills. School administrators were particularly concerned about students skipping lunch and the high rates of food insecurity experienced by families. Students described wanting more emotional supports and feeling overwhelmed.

To address these needs, we adapted activities from a physical activity curriculum previously established in a physical education class‐based healthy lifestyles intervention (Arlinghaus et al. 2021) which followed recommended Supportive, Active, Autonomous, Fair, and Enjoyable (SAAFE) teaching principles to promote physical activity (Lubans et al. 2017) while building social‐emotional competencies. To address social‐emotional needs, we adapted select activities from the Lions Quest social‐emotional learning curriculum (Eisen et al. 2003), an evidence‐based social‐emotional learning curriculum recognized by the National Registry of Evidence‐Based Programs and Practices and the Collaborative for Academic Social and Emotional Learning (CASEL) (Collaborative for Academic Social and Emotional Learning 2015). We consulted with Lions Quest training staff to help determine which activities would be best to integrate into the program and adapted these activities to be more physically active themselves or be augmented with a physically active game. This curriculum was designed to be consistent with recommended Sequenced, Active, Focused, and Explicit (SAFE) practices for social‐emotional learning programs (Durlak et al. 2011; Durlak et al. 2010).

Concerns about food access for families in the district were central in conversations regarding the development of the nutrition education component, reinforcing the importance of providing a nutritious snack or meal after school, which was provided as part of the 21st CCLC. School staff advocated that nutrition education focus on mindfulness approaches to eating rather than weight‐focused approaches, favoring a reduced emphasis on portion sizes and nutrition labels. Mindful eating activities drew from prior mindful eating programs (Kristeller and Wolever 2011) and focused specifically on greater self‐awareness related to internal/external cues for eating and, consistent with the rest of the social‐emotional learning components, also focused on the recognition and regulation of emotions. Table 1 outlines the flow of a typical program session and Table 2 provides an overview of the integrated curriculum, outlining focus points for each social‐emotional competency and how these competencies were integrated with physical and nutrition activities.

**TABLE 1:** Typical program schedule.

Time (minutes)	Activity	Description & rationale
15–20	Welcome game and grounding	Help students transition into afterschool from the school day structure & set positive environment Rapport‐building activities with mentors Mindfulness practices (e.g., breathing exercises, stretching, gratitude, and positive affirmations)
15–20	Mindful eating & snack	Mentors model eating healthy snack Practice mindful eating practices
15–30	Explicit SEL topic	Provide focused time for explicit, sequenced SEL programming & discussion College mentor participation and storytelling increases likelihood students will engage in material Some activities integrated into physical activity (e.g., relays to identify strengths), some are less active (e.g., discussions to practice listening)
30–45	Physical activity	Opportunity to learn & practice physical activity and exercises; mentors model enjoying physical activity and exercise, provide encouragement Choices with varying levels for different skill abilities and variety help support autonomy development. Stations and teams create small groups to promote relationship development Opportunities for active application of SEL topics (e.g., interpersonal development with “copycat” exercises in which one student does a movement and another mirrors that movement; emotion regulation & problem solving to work through naturally arising conflicts like whether a ball is fair play or out)
10–15	Reflection & goal setting	Journaling provides opportunity to explicitly reflect on topics of day Goal setting skills taught & practiced; mentors hold adolescents accountable for goals & help troubleshoot barriers Token economy system for goals and for demonstrating social, emotional, and physical health behaviors taught in DiscoverU.
< 5	Closing	Help transition students to the rest of their day Final mindfulness practices (e.g., gratitude and positive affirmations)

**TABLE 2:** Overview of the integration of social‐emotional learning areas and physical activity and nutrition activities.

Social‐emotional learning area	Focal points	Example exercises/activities
Self‐awareness	Identify values and strengths Recognize emotions & their relation to eating/physical activity Notice how physical activity & eating make you feel Recognize internal vs. external cues to eat Practice eating slowly	Strengths relay race Hunger scale Body check‐ins during and after physical activity Mindful eating meditations Emotions meter
Self‐management	Manage emotions appropriately Use breathing techniques and various types of physical activity to release emotions and find a calm state Set goals and create action plan to achieve them	Breathing exercises Yoga Kickboxing Physical activity & nutrition goal setting discussions
Social awareness	Identify people who can support you to be healthy/adopt health behaviors Identify how social and environmental factors can influence eating and physical activity choices	Role model identification Food advertising stations
Relationship skills	Identify social aspects of physical activity & eating Improve cooperative behaviors and listening skills to make games more fun and manage conflict Resist and respond to negative social pressures	Partner workouts Peer pressure scenarios Affirmation sign race Silent handball Conflict cone ball
Responsible decision making	Use identified values and social supports to make healthy decisions Know positive options for common challenging scenarios Anticipate and evaluate the consequences of eating and physical activity choices	Choose your own adventure problem‐solving skits Walk and shares Anytime anywhere workouts

#### 2.2.5 Stage 2: Development—Mentoring Component

Group mentoring was a key component of DiscoverU. A companion paper (in process) will detail mentor recruitment, training, and outcomes. Briefly, we intentionally recruited 14 college‐age mentors who identified with the racial, ethnic, and gender identities of the adolescents at our partnering school district as this was important to our school partners. While having mentors and mentees with similar gender, racial, and ethnic identities does not necessarily result in better outcomes, mentees often express having mentors they can see themself in is important to them (Blake‐Beard et al. 2011; Koide et al. 2024). Most mentors were current undergraduate students in their third or fourth year (65%). Over half identified as female (57%), and 50% identified as White, with several other racial and ethnic backgrounds represented, including Asian, Hmong‐American, or Vietnamese‐American (21%), Black or Sudanese‐American (14%), and mixed race and ethnicity (Mexican‐American, White; South Asian, White, 14%). Mentors were primarily recruited through university channels (e.g., job boards, announcements in large classes, and activity fairs), and were paid an hourly wage for their time in training and mentoring in the afterschool program.

Mentors participated in 8 h of training pre‐program. Training topics were determined based on discussions between the research team, school staff, and Lions Quest trainers. In the preliminary implementation, mentors were expected to facilitate lessons. As such, initial training emphasized facilitation skills and included 4 h of facilitation skills training from Lions Quest trainers. Additional training topics included training on navigating trauma and adverse childhood experiences from a trauma specialist, and training from research staff on adolescent and youth development and how to talk to youth about nutrition and physical activity. Pre‐program training time was reallocated to reflect a programmatic decision to have separate facilitator and mentor roles following the preliminary implementation. Specifically, mentors' primary responsibility in subsequent implementations of the program was to engage adolescents in program activities and share their own experiences with topics discussed during the program. Accordingly, less training time was spent on facilitation skills and more time was spent on youth development and helping mentors relate to students through storytelling (e.g., explain how they prioritize physical activity in their day or share their own experiences navigating unhealthy peer pressure). We designed training to provide mentors with opportunities to practice skills (e.g., role‐playing and scenario‐based activities). Throughout the program, mentors participated in a 1‐h weekly training designed to be responsive to mentor and programmatic needs observed during the program and recorded using a checklist developed for this study. These weekly sessions included reflection on the prior week, specific topical training as needed (e.g., positive reinforcement), and logistical preparation for the next week. On any given day in the program, there was about a 1‐to‐3 mentor‐to‐adolescent ratio. There was also one afterschool staff person from the school present during each session of the program to help troubleshoot logistical challenges with transportation and be a liaison between school day and afterschool communication.

#### 2.2.6 Stage 3: Program Revisions

The research team continually refined the DiscoverU program based on findings during each implementation iteration. The preliminary implementation of the program began in March 2022, which was the second semester that students were fully back to in‐person learning following the COVID‐19 pandemic. This implementation period also coincided with acts of community violence and threats that led to a couple intermittent instances of afterschool programming being canceled. Research and school staff regularly met and discussed the need to be flexible and patient as the school community underwent the transition back to in‐person learning amidst community tension. Given the necessity for greater flexibility, we recognized major programmatic revisions that needed to be made regarding the dose and schedule, middle and high school integration, mentor role, and some program activities. Following the preliminary implementation, we held separate informal focus groups with mentors, research team members, and school staff. The conversations with mentors and research staff were facilitated and summarized by a consultant. Research team members brought these findings to school staff and discussed ways to improve the program moving forward. Our plan included using the summer months preceding Fall implementation to test some of the planned revisions. We set up a paid internship for high school students to participate in a mini version of the program 1 day/week over the summer and provide feedback on activities and help improve recruitment materials.

Table 3 outlines the specific revisions made at each iteration, the rationale for making each change, and the outcomes of each change. In brief, the preliminary version of the program expected students and mentors to attend two 60‐min DiscoverU sessions each week for 8 weeks. Each week, high school students and middle school students attended the program separately the first day and together for the second day. Each session followed a similar format: group welcome and individual goal check‐in, snack, introduction of the day's topic with brief activity, application of topic through a physically active game, and closing. Main challenges to this initial version of the program included students rarely attended twice a week (on average students attended both days of the week 30% of weeks) and it was logistically difficult to run separate sessions concurrently at two schools. Mentors reported feeling unable to both facilitate and mentor, and research staff noticed they had trouble keeping up with facilitation preparation. Lastly, through observation of lessons and student feedback, it was clear that activities around goal setting were not well liked and were not implemented as designed due to low engagement.

**TABLE 3:** Outline of revisions made to the DiscoverU intervention during development.

	Preliminary Spring 2022 implementation	Fall 2022 & Winter 2023 implementation	Rationale for change	Outcomes
Dose & schedule	60‐min sessions, students expected to attend all 16 biweekly sessions over 8 weeks	120‐min sessions, expected to attend one session per week over 8 weeks	Students rarely attended both sessions in a week	High acceptability of schedule change among adolescents.
				*“[Program leaders] understand if you can't make both days, sometimes if not a lot, at least if you can come a couple of days, they are cool with it.”—Student, Age 14, Winter 2023*
Middle & high school integration	High school and middle school participants met separately at respective schools on day 1 each week, then met together on day 2.	*Fall:* High school and middle school participants met separately for first 3 weeks and together for remaining 5 weeks	Varied attendance made it difficult to prepare high school students to support middle school students; offering concurrent sessions at the middle school and high school was logistically challenging.	Benefits of having middle and high school students together are unclear, and transportation logistics of middle school students walking to the high school may deter middle school student participation.
		*Winter:* High school and middle school participants were together all 8 weeks.		*“I think it's still a little bit of a barrier walking from the middle school for some and might be a deterrent for some. I know there's some parents who don't want, maybe their sixth grader walking up to the middle school or taking the activity bus.”—School Staff, Winter 2023*
Mentor role	College student mentors facilitated sessions and acted as role models	Graduate students facilitated sessions; college student mentors were role models with opportunities for facilitation as ready and interested	Facilitation was challenging and interfered with the ability to focus on the mentoring component.	The role was more appropriate to meet mentors at their skill and interest level.
			*“I did not feel like a mentor, I felt more like a facilitator”—Mentor, Preliminary Implementation*	*“I think for me, the experience definitely improved me in a way that I kinda was able to become a peer leader.”—Mentor, Age 18, Winter 2023*
Goal setting component	Curriculum included a goal setting lesson and 5–10 min each session to check‐in about goals	*Fall:* Goal setting lesson replaced with weekly goal check‐ins	Low interest in goal setting contributed to low engagement. Token economy system suggested by teachers to increase appeal.	Goal setting was acceptable to adolescents and mentors.
		*Winter:* Weekly goal check‐ins plus a token economy system, goal trackers were sent home, and mentors led small groups to plan how to achieve goals each week	*It's hard to keep a goal if you don't even remember what it was—Mentor, Age 20*, Fall 2022	*“I think it [goal setting] really helped them [adolescents] understand that they were like making their goals and participating and just gave themselves a sense of pride, which I think was really good.”—Mentor, Age 22, Winter 2023*

Revisions were made over the summer to address these challenges. To give adolescents more flexibility in attendance while retaining a similar programmatic “dose” we offered the program 2/days week, but extended program time to 120‐min and set the expectation that adolescents attend one session per week for 8 weeks. To streamline logistics, we transitioned to always having sessions with middle and high school students together. Specifically, middle school students walked to the high school each day to engage in the program with high school students. We hired one to two facilitators (i.e., young adult doctoral students) and trained them over the summer to lead activities and manage time and group dynamics, which enabled mentors to do activities alongside adolescents and role model without having to worry about facilitation. Lastly, to increase acceptability of the goal‐setting component, we initiated a token economy system, provided students with take‐home goal tracker cards, and added mentor‐led small group discussions to plan how students would achieve their goals each week.

### 2.3 Formative Evaluation

**FIGURE 1:**
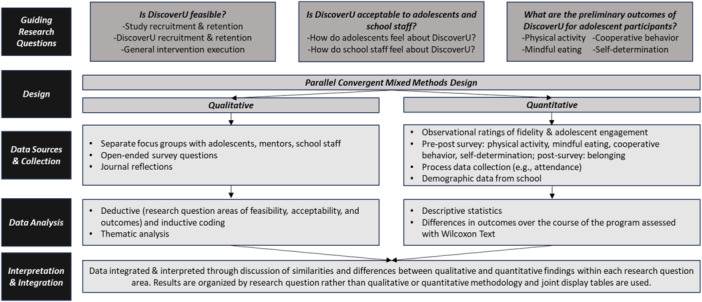
Study design.

Figure 1 outlines the convergent parallel mixed methods design (Creswell and Clark 2017) we used to conduct a formative evaluation of DiscoverU. Quantitative and qualitative assessments were integrated to determine the feasibility, acceptability, and explore the impact of DiscoverU on adolescent outcomes. This mixed‐methods design is ideal for the formative, proof‐of‐concept stage of this research because it provides a more holistic understanding for the feasibility and potential impact of the intervention than only quantitative or qualitative measures alone (Aschbrenner et al. 2022). At this formative stage of intervention development, we are primarily focused on understanding the feasibility of the intervention and study procedures before proceeding to a larger and more cost‐intensive trial. Smaller sample sizes are appropriate for these formative research goals because we are not concerned with generalizability or being statistically powered to detect an intervention effect; instead, we are looking to explore whether there is a signal that the intervention is having the expected effect (Czajkowski et al. 2015).

The district offers afterschool programming on a trimester schedule with 8 weeks of programming each session: Fall (October–December), Winter (January–March), and Spring (March–May). Due to the many program changes following preliminary implementation, our outcome evaluation focused on the Fall 2022 and Winter 2023 implementations of DiscoverU.

#### 2.3.1 Quantitative Measures and Data Collection

Given the feasibility goals of the study, much of the data collected was descriptive in nature. Sources of quantitative data included daily attendance, demographic data on students from the school district, surveys completed by students at the beginning and end of each 8‐week program implementation, and observations recorded by research staff during program implementation every week. Trained research assistants administered and read aloud self‐report surveys, completed by students using paper and pencils. Research assistants told all students they did not have to answer any question that made them uncomfortable. A trained research assistant manually entered survey data into a REDCap survey (Harris et al. 2009) that matched the paper and pencil version. Survey measures included a widely used self‐reported measure of physical activity (Centers for Disease Control and Prevention 2023a; Minnesota Center for Health Statistics 2021), and previously validated pre–post assessments of self‐efficacy to be physically active (Motl et al. 2000), mindless eating and awareness of eating subscales of the Mindful Eating Questionnaire for Children (Hart et al. 2018), cooperative behaviors (Orpinas 1993), self‐determination capacity and opportunity (Wolman et al. 1994), and self‐realization (Wehmeyer 1995), as well as post‐only assessments of belonging (Anderson‐Butcher and Conroy 2002), group cohesion, and group engagement (Kuperminc et al. 2017) (see Tables 5 and 6 for details of response options and alpha coefficients, when appropriate).

#### 2.3.2 Qualitative Measures and Data Collection

Participant and partner groups, including adolescents, mentors, facilitators, and school staff provided formal qualitative data. We conducted focus groups following program implementation with adolescent participants (Winter 2023: *n* = 6 middle school students and *n* = 8 high school students) and with mentors (Fall 2022: two groups of *n* = 7 each; Winter 2023: two groups of *n* = 5–6 each), facilitators (end of Winter 2023 only, *n* = 3), and school staff (end of Winter 2023 only, *n* = 3). Focus groups with adolescents and mentors were slightly shorter (~30–45 min) than those with facilitators and school staff (~60–75 min). Focus groups by the co‐principal investigators (adolescent, mentor, and school staff) and a senior co‐investigator (facilitators).

Focus group questions were complementary to one another with questions aimed at exploring the feasibility and acceptability of the intervention and to contextualize program attendance and quantitative survey data from the varying perspectives of involved parties. For example, student focus group questions included: “What are you doing after school, if you are not here at DiscoverU?,” “Who do you talk to about DiscoverU and how do you describe it?,” and “How are you using things we do at DiscoverU outside of the program?” A multi‐part question for mentors was, “What are some new skills and experiences that you are taking away from DiscoverU, such as job/professional skills or something you've learned more about yourself personally and taking care of your health?” Example questions for facilitators included, “What is your favorite memory about DiscoverU?” and “Please talk through a challenge or barrier that you overcame, in your role as a DiscoverU facilitator.” Example questions asked of school staff included “How would you describe the evolution of the DiscoverU program since it started?” and “In what ways (if any) do you think the people involved in DiscoverU or the afterschool environment have changed since DiscoverU was implemented?” We audio‐recorded and transcribed each focus group verbatim. Other sources of qualitative data included open‐ended response questions on student surveys (e.g., “why did you join DiscoverU?”) and program journals which prompted adolescents and mentors to reflect on the program at the end of each session.

### 2.4 Analysis Strategy

#### 2.4.1 Quantitative Analysis

We computed descriptive statistics for all quantitative data. We pooled attendance and pre–post survey data from Fall and Winter implementations because program changes made between the two implementations were minimal. In the instances in which an adolescent participated in both Fall and Winter implementations (*n* = 14), we included their Fall survey data (baseline) in the pooled analysis. We conducted Paired Wilcoxon (signed‐rank sum) tests to assess pre–post changes in outcomes. Due to the small sample size and exploratory nature of this pilot study, we used nonparametric tests and a *p*‐value of < 0.10 to indicate statistical significance.

#### 2.4.2 Qualitative Analysis

All qualitative data were merged into one dataset for analysis. This approach is frequently used in pragmatic implementation studies and is appropriate for our stage of intervention development (Ramanadhan et al. 2021). Three research team members (MO, KA, and LG) conducted deductive and inductive thematic analysis to identify themes (Braun and Clarke 2006). The three qualitative analysts were all female, trained in qualitative methods (i.e., had taken one or more graduate‐level qualitative methods classes), and had been actively involved in the development and implementation of the program. The analysts had different levels of experience and education: two were registered dietitians; one had a master's in public health and was current epidemiology doctoral student; one had a bachelor's degree in psychology and was a current dual master's of public health and master's of social work student; and one had a PhD in Kinesiology. The analysts all independently completed first‐level, deductive and inductive descriptive coding. Deductive codes included each evaluation area of interest (feasibility, acceptability, and impact). Team members discussed inductive codes to create a consensus codebook. Two research team members (MO and LG) used the consensus codebook to independently code all focus group transcripts and journal entries. Minor coding disagreements were identified and reconciled in a series of meetings with all three analysts. Once all codes had been agreed upon, the three analysts independently memoed to identify patterns between inductive codes within each deductively coded research area. These patterns were discussed to identify and agree upon themes. The coding tree is provided in Supporting Information Table S1.

#### 2.4.3 Integration

Following data analysis, the research team met several times to identify and interpret similarities and differences between data sources. Like the qualitative data, the quantitative findings were organized by area of investigation (i.e., feasibility, acceptability, and impact). Related qualitative and quantitative data were paired in a joint displays (e.g., quantitative metrics for acceptability were paired with qualitative acceptability themes), allowing for direct comparison between the quantitative and qualitative findings and synthesized in the narrative through weaving (Fetters et al. 2013). This integration of qualitative and quantitative data provided both confirmation (agreement of results across the two types of data) and expansion with the qualitative data providing additional context to the quantitative data.

## 3 Results

### 3.1 Feasibility: Recruitment & Retention

Results of qualitative and quantitative feasibility data confirmed one another, with qualitative data providing additional context that expanded our insights regarding the feasibility of program. We identified three qualitative themes related to feasibility: (1) DiscoverU appealed to a diverse group of students with health‐related and social needs; (2) social relationships facilitated recruitment and retention; and (3) communication and flexibility were key to coordinating a program that involves so many different roles. These themes are explained below, weaved into the quantitative process findings regarding student recruitment, retention, and program execution to illustrate how the qualitative findings confirm and expand the interpretation of quantitative findings.

#### 3.1.1 Recruitment

Table 4 provides demographic characteristics of consented participants (*n* = 32). Participants identified with a diverse range of race/ethnicities (38% Black or African American; 28% Hispanic/Latino; 16% Asian; 9% White; 9% two or more races) and 88% were eligible for free or reduced‐price school meals. DiscoverU participants tended to be female (78%) and high school students (66%) rather than males and middle school students. Respectively, 25% and 29% of students screened positively for symptoms of depression and anxiety, and 23% reported having a health condition that makes it hard to do some things others their age can do. These characteristics aligned with the first qualitative theme that DiscoverU appealed to a diverse group of students with health‐related and social needs. For example, afterschool staff described conversations they overheard during program,

The kids are pretty open about some of the things that they we're going through like….talking about their gender dysphoria, and like trying to figure out who they wanted to be…Some kids would talk about anxiety….somebody has ADHD…kids talking about learning disabilities…or mental health things that they are dealing with…or with a lot of students with food disparities and like below the poverty line that were involved that would talk about like, “Well, this snack is my supper.”

**TABLE 4:** Descriptive characteristics of consented DiscoverU students (*N* = 32, Fall 2022 and Winter 2023).

	Mean (SD) or count [%]
Age	14.8 (1.97)
School grade	
Middle (6–8)	11 [34%]
High (9–12)	21 [66%]
Sex	
Female	25 [78%]
Male	7 [18%]
Gender identity	
Transgender, genderfluid, genderqueer, nonbinary, or unsure	6 [23%]
Cisgender	13 [59%]
Unsure about question	3 [14%]
Race	
Asian	5 [16%]
Black	12 [38%]
Hispanic/Latino	9 [28%]
White	3 [9%]
Two or more races	3 [12%]
Home language	
English	9 [53%]
Hmong	3 [9%]
Italian	1 [3%]
Somali	3 [9%]
Spanish	9 [28%]
Tibetan	1 [3%]
Free/reduced priced lunch status	
Not eligible	2 [6%]
Free/reduced	30 [94%]
Physical or health condition	
No	20 [77%]
Yes	6 [23%]
PHQ‐2 depressive symptoms	
Negative screen	21 [75%]
Positive screen	7 [25%]
GAD‐2 anxiety symptoms	
Negative screen	20 [71%]
Positive screen	8 [29%]

*Note:* Missing data were present for adolescent reports of gender identity (*n* = 10); physical health condition (*n* = 6); depression (*n* = 4); and anxiety (*n* = 4). PHQ‐2 is the Patient‐Health Questionnaire‐2 screener (Kroenke et al. 2003). GAD‐2 is the Generalized Anxiety Disorder‐2 screener (Kroenke et al. 2007).

The second qualitative theme related to feasibility was that social relationships facilitated recruitment and retention. The most beneficial recruitment strategy was when college mentors came to the school (i.e., at lunch, before school, or visits to health classes) to engage students in learning about the afterschool program. Recruitment was particularly successful when college‐age mentors approached students rather than rely on student initiative to come to the mentors. Teacher promotion of afterschool activities was also helpful, but inconsistent. School administrators partially attributed declines in overall afterschool program participation to reduced teacher participation,

We're not getting as much teacher buy in…We're even hiring some aids and such to run some [afterschool] classes which is great. But then we miss that teacher promotion of you know, really talking it up and getting kids there, which, when they have a really fun class and teachers do a good job of promoting it you can see that [in student attendance].

Students reported joining DiscoverU because they wanted to meet the college‐age mentors. For example, one adolescent explained, *“[I joined DiscoverU] for a nice look at college [life] and make friends.”* Students themselves supported recruitment through word‐of‐mouth and by inviting their friends to attend. Although students did not specifically say they came to the program for the snack, students' descriptions of DiscoverU consistently included an emphasis on the snack that was provided. For example, one adolescent explained how they described DiscoverU to friends, *“We have snacks here. She's like ‘really?’ I'm like, come if you want it. [lots of noise, agreement from everyone in focus group]. So, if you want snacks, come with me.”*

This type of word‐of‐mouth recruitment resulted in some students beginning the program a few weeks into the program. A total of 48 students attended at least one session in Fall 2022, 25 (52%) of whom consented to participate in data collection for this study. Of these 48 students, 10 of them started attending in week 3 or later in the program. A total of 29 students attended at least one session in the Winter, 22 (76%) of whom consented to participate in data collection for this study. Of the 29 Winter students, six started attending at week 3 or later in the program. Over the course of the Fall and Winter implementations, a total of 32 unique students consented to participate in data collection,14 of whom (44%) participated in both sessions.

#### 3.1.2 Retention

**FIGURE 2:**
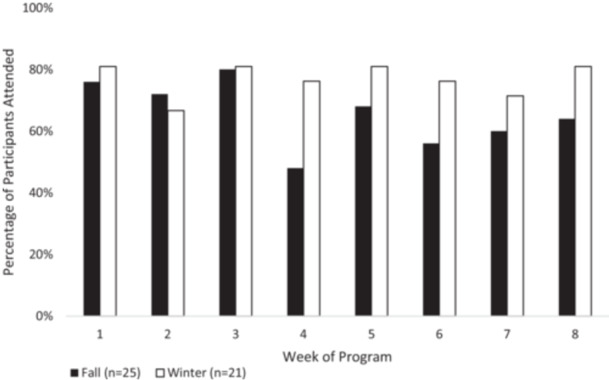
Participant attendance by program week, *N* = 32 unique adolescents (14 participated in both Fall and Winter implementations).

Figure 2 illustrates the percentage of participants that attended each week of program. Generally, each week 70%–80% of participants attended program; however, attendance was inconsistent on an individual basis. Respectively, 44% and 52% of adolescents attended at least one of the days offered each week for 7–8 weeks of the 8‐week program in Fall and Winter. Attendance consistency improved from Fall to Winter implementations, with the percentage of participants who only attended 1–2 weeks falling from 16% in the Fall to 0% in the Winter.

Despite reports of joining DiscoverU to improve their mental health, students also reported missing the program due to poor mental health or being mentally exhausted after the school day. For example, one adolescent explained, *“[I need to] go home and do my own thing after school because pretty much like I'm exhausted.”* In addition to not being mentally up for staying after school, students reported having responsibilities at home that prevented them from attending. For example, one student described,

If I'm not coming to DiscoverU, I go home and help babysit…do household chores…groceries for my parents. I get sent off to do that by myself. So I'm a big help out with the house. Sometimes, I'm just so mentally tired….I just have to go home and reflect on what caused it and how I can fix it. And sometimes we have a crisis in the family when like something bad happens.

Some students mentioned missing DiscoverU due to competing afterschool activities (i.e., different clubs), but often students would stop by before or after the program to still connect with the mentors. Students also reported missing DiscoverU because of schoolwork (e.g., upcoming test). Occasionally, students, would do their homework at DiscoverU and not participate in specific activities. College‐age mentors informally provided homework assistance before and after the program. Even when students did not attend DiscoverU, they often sought contact with mentors to continue social connections. A facilitator explained,

A lot of times we'll have kids who will just like pop in…they just want to let us know that they can't make it today, but they wanted to…and it was like a lot of different kids. It wasn't just one specific kid. Most of our kids who come all the time would be like I'm sorry I can't make it, I'm like behind on my math test but I would love to be here, might be able to come later.

#### 3.1.3 Program Execution

The final qualitative theme related to feasibility identified that communication and flexibility were key to coordinating a program that involves so many different roles. School and university staff appreciated one another's flexibility and shared the expectation that afterschool programming is dynamic. One school staff member explained,

I feel like sometimes it was a little chaotic, as afterschool programming can be like. “Wait, what do you mean? The door is locked. You can't get it. I reserved it…” so it's always something. I feel like we were putting out some little fires all the time, but as much as you plan for everything you can. Some of that stuff still pops up, so I also appreciate [the University team's] flexibility, and just going with the flow and working our way through some of those things.

Facilitators also described how they had to be responsive to the dynamic nature of afterschool programming, *“you have to be a good juggler”* and *“when you're actually in program you have to be responsive to changes and improvise and try to keep things going.”* Overall, school staff reported feeling that *“everything ran structurally really well,”* and reflected on the improvements that had been made overtime as the program was developed and revised. School and university staff alike noted that communication across the many parties involved (e.g., custodial staff, teachers, facilitators, parents, mentors, and research staff) was important to smoothly execute the program and research activities.

### 3.2 Acceptability

DiscoverU was highly acceptable across all involved parties. All students (100%) reported that they would recommend DiscoverU to a friend. As seen in Table 5, students reported feeling comfortable (76.9%) and accepted (84.6%) in DiscoverU, and that they found the activities interesting (80.8%). This high level of acceptability was confirmed by qualitative findings. We identified two themes related to acceptability from qualitative data, which both confirmed the quantitative acceptability findings and further contextualized the high level of satisfaction and belonging: (1) social connections and the mentoring component made DiscoverU enjoyable, and (2) DiscoverU created a safe, inclusive environment for student and mentor participants to be themselves and explore new activities and friendships. These themes are described below to expand on the quantitative acceptability ratings. Additional exemplar quotes are provided in Table 5 alongside the quantitative acceptability metrics to provide a joint display of the integrated quantitative and qualitative findings related to acceptability.

**TABLE 5:** Acceptability results from post‐program quantitative and qualitative analyses (Fall 2022 and Winter 2023).

Acceptability metrics	*n*	% rated very much	Acceptability exemplar quotes from adolescent participants
Belonging			“They [Mentors] made me feel good because I could be sitting alone, [mumbles]. It's nice…We have something to talk about…Plus they're like really nice.”—Student, Age 15
I feel comfortable at DiscoverU.	26	76.9%	
I am a part of DiscoverU.	26	84.6%	
I am committed to DiscoverU.	26	69.2%	“They [Mentors] make me feel very happy. They're always positive and kind. They support me. They give me confidence too; tell you can do this, you got this”—Student, Age 15
I am supported at DiscoverU.	26	72.0%	
I am accepted at DiscoverU.	26	84.6%	
Group cohesion			“Working as a group that we can achieve a lot of things.”—Student, Age 15
People in DiscoverU care about each other.	25	76.9%	
People in DiscoverU make each other feel good.	26	80.0%	“They [mentors] made me feel good about myself. I feel like whenever I see them, it seems like positive vibes.”—Student, Age 17
When someone says something, it stays at DiscoverU (nobody will repeat it outside the program).	26	69.2%	“Last season, I went through a hard breakup and I talked with the mentors about it and they all gave me great advice on how to handle it. And I very appreciate it because I've done well with it… I was with my DiscoverU family.”—Student, Age 14
If people in DiscoverU are really mad or upset about something they can talk about it at DiscoverU.	26	57.7%	
Group engagement			“I love today! We workout; it was so fun! Can't wait to go tomorrow.”—Student, Age 12
When you are at DiscoverU, how much do you enjoy the activities you participate in?	26	65.4%	“This was a 10/10, will try this at home. The people here are nice. I feel like I belong here. Mindful eating was a first time for me. I think it's good for me cause there will be lots of things on my mind.”—Student, Age 12
Do you think the activities you do at DiscoverU are interesting?	26	80.8%	

*Note:* Quantitative post‐program survey data were pooled from Fall 2022 and Winter 2023 for *n* = 32 unique adolescents; missing data on these survey items were present for *n* = 6 students. Qualitative data were collected during Winter 2023. Response options for acceptability survey questions ranged from 0 “Not a lot” to 3 “Very Much.” Items measuring belonging were adapted from the Sense of Belonging scale (Anderson‐Butcher and Conroy 2002). Items measuring group cohesion and group engagement were adapted from a Group Mentoring Climate scale (Kuperminc et al. 2017).

First, social connections and the mentoring component made DiscoverU enjoyable. Students, mentors, and facilitators reported enjoying engaging in program components together. When students reflected on an activity, their reflection usually also mentioned an aspect of the social connection that occurred through the activity. For example, one adolescent wrote in their journal, *“Today was very memorable because we had fun playing dodgeball and it was very competitive. I laughed a lot, and it was nice.”* School staff described the program as, *“It's…a place where you can come and ask those questions and get that advice that you might not be able to get at home or get at school.”* This quote illustrates how social connections developed in DiscoverU were different from other social connections the students may form elsewhere. Mentors and facilitators also reported enjoying developing social connections with adolescents through engagement in activities and sharing about each other's lives. For example, one mentor said, *“I connected with [adolescent] because…He opened up to me about several things going on in his life. We enjoyed competing with each other during activities and making jokes!”* Reports of disliking program components were rare and due to individual preference. For example, one participant reported they preferred a different grouping strategy when developing teams for activities.

Second, participants felt like they belonged because DiscoverU created a safe, inclusive environment for student and mentor participants to be themselves and explore new activities and friendships. By design, participants (mentors and students) met new people across age groups while having conversations that encouraged reflection upon personal topics. DiscoverU's cross‐age mentoring component fostered mutual vulnerability that promoted community, social support, and an environment where students felt included, safe, and accepted. For example, one DiscoverU student described how mentors helped them to engage in the physical activity of the day which was often strenuous for students, *“They… encourage you to, like, let's say it's a hard exercise that we're doing… Like, ‘Oh, you can do this…’ and then do it with you. And that might or may not like give you more strength and confidence.”* The verbal encouragement, modeling, and engaging in exercises with students described by the student were all heavily emphasized responsibilities of college students in their role as mentors. The supportive environment was well received by students and set the stage for participants to being willing to try new activities and learn from each other. Facilitators also acknowledged how support was a key aspect of their role, “*why we really do this, is to listen to them and hear them say these, you know, really insightful things that they're learning and exploring themselves, and we're really just there to be with them and help them support that.”* This support was well‐liked by students. School staff also expressed appreciation for DiscoverU's supportive environment to foster adolescent growth, particularly following the COVID‐19 pandemic. For example, one staff member said, *“Especially with some of our students, and coming out of the pandemic, and just like the way it is now, I feel like… we're serving very much of a need for students.”*

### 3.3 Initial Outcomes

Table 6 provides a joint display of quantitative and qualitative findings for direct comparison. Specifically, quantitative pre–post scores on self‐reported physical activity, mindful eating, and social‐emotional related skills. Except for the awareness of eating subscale, all metrics moved in the expected positive direction, however, this movement was only statistically significant from pre‐program to post‐program for two outcomes: physical activity, which increased by 1 day per week (*p* < 0.01), and cooperative behaviors (*p* = 0.05). One qualitative theme related to impact was identified: Social connections established in DiscoverU helped facilitate social‐emotional skill development, physical activity, and positive eating behaviors. This theme is described below. It expands upon the quantitative data by indicating that changes seen in physical activity and cooperative behaviors were likely due to the social connections established in the program.

**TABLE 6:** Initial outcome results from quantitative and qualitative analyses (Fall 2022 and Winter 2023).

Outcome metrics	*n*	Pre‐program mean (SD)	Post‐program mean (SD)	*p* value	Outcome exemplar quotes from adolescent participants
Physical activity					“My team and the other team were a sweat. I've never ran like this before. I felt burn in my chest. But in the end I very much enjoyed it! Then we also did some breathing to calm ourselves down.”—Student, Age 17
PA days in a week	21	2.95 (1.94)	4.00 (1.87)	**< 0.01**	
PA self‐efficacy	23	3.16 (0.80)	3.35 (0.95)	0.10	
Mindful eating					“One thing I do is mindful eating, so like sometimes…when I'm eating something I realize that I'm just like not even paying attention. Like when I'm watching a movie or something. And I think back to Discover U and be like, let me like breathe or like take a moment before I continue eating because I don't want to be too full later.”—Student, Age 15
Mindless eating	23	2.83 (0.56)	2.57 (0.55)	0.19	
Awareness of eating	23	3.80 (0.54)	3.60 (0.81)	0.21	“I try to have connections to what I'm eating because I have a snack drawer in my bedroom but like nothing in there is healthy… it's like pop, cookies… ok all this unhealthy stuff. And I kinda like hey, do I really want this…. And then I'll put it away…it just made me think about it just doesn't fill me up, it's just eating but when I eat fruit I feel a difference, like I'm not hungry any more. I think that the difference is like from when we learned from [DiscoverU facilitator]”—Student, Age 14
Social‐emotional skills & self‐determination					
Cooperation with peers	23	5.09 (1.38)	5.74 (1.25)	**0.05**	“Though I came late, we played handball. It was fun. Everyone had great teamwork!”—Student, Age 16
Self‐determination capacity	23	14.0 (4.31)	14.7 (3.92)	0.36	“My problem‐solving skills…friend A was angry at friend B because friend B said some questionable things to her, I was obviously on friend A's side because friend B was wrong. I helped friend A calm down and eventually, friend A regained friend B.”—Student, Age 15
Opportunities for self‐determined behaviors	23	15.0 (4.98)	15.5 (5.16)	0.70	“I feel like I do ‘feel, think, do’ a lot more than I used to. When I first started DiscoverU, we did talk about it. I brought stories about the things I did, which were not okay, and I could have done better for how I reacted. And what I learned with ‘feel, think, do’ is I've been more in control of my emotions, especially when there's conflict. Instead of me bursting about trying to find the simple way out…”—Student, Age 14, Multiracial
Self‐realization	23	3.51 (0.71)	3.62 (0.73)	0.31	

*Note:* Quantitative post‐program survey data were pooled from Fall 2022 and Winter 2023. Qualitative data were collected Winter 2023. PA = physical activity. The item measuring days physically active for a total of at least 60 min per day was adapted from the CDC YRBSS (Centers for Disease Control and Prevention 2023a); responses of 1 “yes” were counted across the last 7 days. Physical activity self‐efficacy was a 4‐item mean‐averaged scale (alpha = 0.71 at baseline) adapted from the physical activity self‐efficacy questionnaire (Motl et al. 2000); response options ranged from 1 “Disagree a lot” to 5 “Agree a lot.” Mindless eating was an 8‐item mean scale (alpha = 0.48 at baseline) and awareness of eating was a 4‐item mean scale (alpha = 0.53 at baseline) from the Mindful Eating Questionnaire for Children (Hart et al. 2018); response options ranged from 1 “Disagree a lot” to 5 “Agree a lot.” Questions measuring cooperation with peers were adapted from a skills training for violence prevention in middle school (Orpinas 1993) and created by the research team; responses of 1 “yes” were counted across seven questions. Self‐determination capacity and opportunities for self‐determined behaviors were both 6‐item mean‐averaged scales (alphas = 0.86 and 0.84 at baseline, respectively) from the AIR Self Determination Scale (Wolman et al. 1994); response options ranged from 0 “Never” to 4 “Always.” Self‐realization was a 10‐item mean‐averaged scale (alpha = 0.85 at baseline) from the ARC Self‐Determination‐Adolescent Version (Wehmeyer 1995); response options ranged from 1 “Disagree a lot” to 5 “Agree a lot.” *P*‐values were obtained using a paired Wilcoxon (signed‐rank sum) test. Bolded values indicate statistical significance at *p* < 0.10 or less.

Qualitative data from across focus groups expands quantitative data, which did not assess the development of social connections as an outcome, by indicating that DiscoverU fostered social connection between adolescents. For example, one adolescent said, *“There are some people in my classes that I've never talked to until DiscoverU so that's a fun thing that we've learned…to actually talk to other people and be more open‐minded about it.”* A facilitator reported, *“[Adolescent] and [adolescent] didn't know each other before DiscoverU … now they're like really besties, and it's just been really fun to watch… [they] definitely became really good friends, and I think DiscoverU played a role in that.”*

Adolescents and mentors further described how supportive mentorship may have led to the improvements in cooperative behaviors by helping students problem‐solve conflicts in their life that they didn't want to discuss with parents. One adolescent said, *“Well I'm the oldest so I don't have an older sibling or someone to talk to. So it's kinda necessary to talk to them [the mentors]. Like the things you don't wanna tell your parents but you still need someone to talk to.”* Additionally, one mentor reported in their journal, *“I loved seeing how the kids from the high school and middle school were interacting. It was awesome to see their teamwork skills developing. I want to remember how the students were motivating one another.”* Table 6 provides additional exemplar quotes on how social connections established in the program impacted physical activity and eating behaviors. Consistent with this finding is that many of the physical activities done in the program were group games (e.g., handball and cone ball) or partner workout activities which both facilitated students to be active and required cooperative behaviors and teamwork.

Despite the positive reports of engaging in promoted health behaviors, it is unclear whether the support provided during DiscoverU was sufficient to extend beyond the program setting. School staff noticed that some adolescents would *“come out of their shell”* in program, but they would revert to being less open during the school day. However, some adolescents reported applying the skills they learned in DiscoverU and implementing advice they received from mentors outside of program. For example, adolescents reported, *“Yeah, I use the values bracket often now, every time I make a decision now I think, what are the values that I have that are important to me”* and, *“Yeah like, it made me focus on food or focusing on doing exercise, I feel like it just helps me encourage myself more. Like maybe I can do this. It encourages me to do more of the stuff we do in DiscoverU.”*

## 4 Discussion

The purpose of this paper was to describe the formative development of the DiscoverU mentoring program and to use a convergent mixed‐methods approach to evaluate program feasibility (e.g., attendance and program execution), acceptability, and impact on adolescents' physical activity, interpersonal skills (e.g., cooperative behaviors) and social connection with mentors. To our knowledge, DiscoverU is the first afterschool program to use cross‐age, group mentoring to integrate social‐emotional learning, physical activity, and nutrition education for adolescents, with specific attention to school systems and ecology (i.e., key tenets of community psychology; Trickett and Rowe 2012). DiscoverU reached an ethnically and racially diverse group of middle and high school students with multiple health‐related needs, was highly acceptable, and illustrated some promising initial outcomes in social connection (i.e., belonging), self‐reported physical activity, and cooperative behaviors. However, additional revisions to DiscoverU could improve feasibility, as only about half of participants attended 7–8 weeks of the program.

The high proportion of adolescent students who reported anxiety and depression symptoms in our sample aligns with 2021 findings from the Youth Risk Behavior Surveillance System in which 42% of U.S. high school students reported persistent feelings of sadness or hopelessness (Centers for Disease Control and Prevention 2023b). Coming out of the COVID‐19 pandemic, afterschool time is increasingly viewed by schools and families as an opportunity to supplement the school day to help students “catch up” academically and socially. In the present study, school staff were excited to offer an afterschool program that could help address some of the health needs of students. Qualitative findings suggest that efforts to support adolescent health are needed in multiple settings. We found some adolescents' mental health prevented them from attending the afterschool program. After a demanding school day, students often reported wanting to go home to relax. The push to have youth do more while struggling with their mental health may be counterproductive. As reflected in our iterative program refinement process, we found changing the expectation of attending the program from 2 times to 1 time/week was more manageable for adolescent participants, but even with this change only about half of students attended the full program. Creative ideas are needed to address these overlapping issues of having an intensive enough program to be meaningful, but not so intensive that it prevents students from attending. This may be especially important for adolescents, who typically have more independence and autonomy when it comes to attendance compared to elementary school students. Further research is needed to identify solutions to improve consistent attendance. Given how well‐liked the mentors were, it could be possible to lean into this strength of the program to boost attendance. Other ideas could include integration of additional mental health services beyond the universal social‐emotional learning curriculum, particularly for those students already struggling with mental health.

Despite the inconsistent individual attendance, participants felt a high sense of belonging at DiscoverU, reported high group cohesion, and high group engagement. These outcomes are promising as belonging and social support are important protective factors for isolation and suicide risk. Notably, the cross‐age mentoring component of the program was a key reason for the high acceptability and high sense of belongingness. Participants reported feeling supported by mentors and thought of them as older brothers and sisters. Fostering social connectedness is a crucial aspect of developmental mentoring, which aims to lower the number of youth engaging in risky health behaviors by drawing youth in to participate and grow their affection for the people and communities in their lives (Jessor and Jessor 1977; Karcher 2005). Given the important role mentors played in the adolescent outcomes and acceptability of the program, a separate paper in progress will delve into the feasibility of sustaining the high‐quality group mentoring component.

In focus groups, adolescents cited the mentoring component as an essential motivation to be physically active during program and reported how mentors supported them to keep going during physical activity. This finding indicates that mentoring served as the “supportive” criterion in the SAAFE principles to promote physical activity (Lubans et al. 2017). Self‐reported pre–post gains of about 1 day of physical activity per week are consistent with youth attending DiscoverU about 1 day/week. This suggests that DiscoverU may have increased PA on programming days, but not overall, or outside of structured intervention time, similar to what has been reported in other afterschool PA interventions (Mears and Jago 2016; Pate and O'Neill 2009; Sliwa et al. 2023; Tapia‐Serrano et al. 2023). The inability of these interventions to increase physical activity outside of set structured opportunities highlights the value of identifying effective strategies to increase enrollment and regular attendance in this type of programming.

Overall, our analysis of outcomes found that significant changes in cooperative behaviors and time spent being physically active were in the expected direction, indicating DiscoverU's integrative approach may have the potential to make positive impacts across multiple adolescent health domains. Positive changes in cooperative behaviors alongside the promising increases in self‐report of physical activity are consistent with our program's expectations, as many active games played required participants to engage in cooperative behaviors to play successfully.

School staff advocated for a mindful eating approach in nutrition education, noting the importance of promoting positive eating behaviors with *any* food to help overcome supply chain issues that influenced snack options and to avoid promoting foods that students may not have access to at home. Adolescents enjoyed mindful eating and found it novel. Given the varied attendance rates, a subset of participants may have needed more exposure to see statistically significant positive changes in both the mindless eating and awareness of eating subscales. While mindful eating is well‐aligned with the social‐emotional construct of self‐awareness, the impact of mindful eating on dietary intake, especially among youth and populations experiencing food insecurity, is unclear at this time (de Lara Perez and Delgado‐Rios 2022; Tapper 2022).

### 4.1 Strengths and Weaknesses

Several strengths characterize this study. The development of DiscoverU was grounded in theory (i.e., social cognitive theory, Bandura 2004) and best practices for community‐engaged interventions, explicitly taking into account contextual and systemic factors such as community input and root causes of disparities. The mutually beneficial partnership between the University and local school district and collaborative planning helped ensure the program was feasible to implement, aligned with school and participant needs, and contributed to community development. The mixed methods study design and data collection across all involved parties provided a thorough understanding of the feasibility and acceptability of the program. The use of validated and/or widely used measures is another strength of the evaluation.

Our study is limited in that we did not collect detailed demographic data on the facilitators or school staff who participated in focus groups, mainly due to the risk of breaking confidentiality and identifying particular individuals. The one‐group design and small scale (single district and small sample) are appropriate for the design phase of the intervention. However, not having a comparison group and statistical power limits our understanding of the impact the intervention had on adolescents' physical activity, mindful eating, and social‐emotional skills. The results of this demonstration project cannot be generalized to other populations and may be subject to selection bias, given the small sample size and that roughly half of DiscoverU program participants enrolled in the study component. The cross‐age mentoring strategy used in this study could be transferred to other programs taking into careful consideration the logistics of a university being near the K12 district and the University having the resources to put on the program so that minimal daily work was required on the part of the school to facilitate the program.

## 5 Conclusions

Coming out of the COVID‐19 pandemic, afterschool programs are increasingly being looked upon by public health professionals and schools to help adolescents overcome academic deficits, as well as declines in mental and physical health. Many afterschool programs serving low‐income communities continue to be challenged by unstable resources and high student health needs in a post‐pandemic environment. Relevant to the field of community psychology, our findings highlight how afterschool practitioners could think about partnerships with local universities as a strategy to help supplement the capacity of programs and school staff by providing additional caring young adults to support and make health appealing to adolescents in afterschool programming. The DiscoverU afterschool group health mentoring intervention was highly acceptable by all involved parties and successfully promoted a sense of belonging and physical activity engagement among a diverse group of adolescents. Notably, we found that the group mentoring component of the program was a key strength that related to both adolescent acceptability and outcomes. These proof‐of‐concept findings indicate that the cross‐age, group mentoring model developed here warrants additional research and consideration by practitioners as a promising empowerment strategy to make universal health programming appealing to adolescents.

## Author Contributions

**Katherine R. Arlinghaus:** conceptualization (equal), writing – original draft (lead), formal analysis (equal), writing – review and editing (equal), funding acquisition (equal), investigation (equal). **Kristen Stuenkel:** writing – review and editing (supporting), conceptualization (supporting). **Jodi Gadient:** writing – review and editing (supporting), conceptualization (supporting). **Adrianna N. Bell:** writing – review and editing (supporting), formal analysis (supporting), investigation (supporting). **Lenora P. Goodman:** writing – review and editing (supporting), formal analysis (supporting), investigation (supporting). **Nancy E. Sherwood:** conceptualization (supporting), writing – review and editing (supporting). **Barbara J. McMorris:** conceptualization (equal), formal analysis (equal), writing – review and editing (equal), funding acquisition (equal), investigation (equal).

## Ethics Statement

This research was approved by the University of Minnesota Institutional Review Board (#00015412) on March 22, 2022. All adult participants provided informed written consent and all child participants provided informed written parental consent and written child assent.

## Conflicts of Interest

The authors declare no conflicts of interest.

## Supporting information


Supporting File

## Data Availability

The data that support the findings of this study are available from the corresponding author upon reasonable request.
