# Supplementary material for: “DiscoverU”: Formative Development and Mixed‐Methods Evaluation of an Afterschool Health and Well‐Being Mentoring Program for Adolescents
Source: J Community Psychol. 2026 Aug 5;54(6):e70137. doi: 10.1002/jcop.70137 (PMC13440985; doi:10.1002/jcop.70137)
Supplement: Supplementary file 1 [file JCOP-54-0-s001.docx]

Supplementary Material

| **Supplementary Table 1. Qualitative Coding Tree** | | |
| --- | --- | --- |
| **Research Area (Deductive Code)** | **Inductive Codes** | **Themes** |
| Feasibility | Sample population | DiscoverU appealed to a diverse group of students with health-related and social needs |
|  | Family circumstances |  |
|  | Recruitment | Social relationships facilitated recruitment and retention |
|  | Attendance |  |
|  | Communication | Communication and flexibility were key to coordinating a program that involves so many different roles |
|  | Coordination |  |
| Acceptability | Connection-activity | Social connections and the mentoring component made DiscoverU enjoyable |
|  | Connection-mentors |  |
|  | Connection-general |  |
|  |  |  |
|  | Challenge | DiscoverU created a safe, inclusive environment for student and mentor participants to be themselves and explore new activities and friendships. |
|  | Enjoyment |  |
|  | Social climate |  |
|  | Support |  |
| Impact | Social connection | Social connections established in DiscoverU helped facilitate social-emotional skill development, physical activity, and positive eating behaviors. |
|  | Social confidence |  |
|  | SEL skill growth |  |
|  | Physical activity self-efficacy |  |
|  | Eating awareness |  |
